# Carbon dioxide angiography during peripheral angioplasty procedures significantly reduces the risk of contrast-induced nephropathy in patients with chronic kidney disease

**DOI:** 10.1186/s42155-020-0103-z

**Published:** 2020-02-17

**Authors:** Athanasios Diamantopoulos, Lorenzo Patrone, Serafino Santonocito, Iakovos Theodoulou, Shazhad Ilyas, Renato Dourado, David Game, Narayan Karunanithy, Sanjay Patel, Hany Zayed, Konstantinos Katsanos

**Affiliations:** 1 Department of Interventional Radiology, Guy’s and St. Thomas’ Hospitals, NHS Foundation Trust, London, SE1 7EH UK; 2 Kings College London, School of Medicine, London, UK; 3 Department of Vascular Surgery, 1st Floor, North Wing, Westminster Bridge Road, London, SE1 7EH UK; 4 Department of Nephrology, Guy’s and St. Thomas’ NHS Trust, 6th Floor Borough Wing, Guy’s Hospital, Great Maze Pond, London, SE1 9RT UK

**Keywords:** Contrast media, Angioplasty, Carbon dioxide, Acute kidney injury, Endovascular procedures, Contrast induced nephropathy

## Abstract

**Background:**

Iodinated contrast media are amongst the most frequently prescribed medications, however, their use is not without complications. With contrast-induced nephropathy constituting a major concern, alternative non-iodine based approaches have been explored such as carbon dioxide angiography. The purpose of this study is to report the incidence of contrast-induced nephropathy following carbon dioxide angiography in patients with impaired renal function that underwent peripheral angioplasty compared with a historical cohort of patients that underwent angioplasty with use of solely iodine contrast medium. The historical cohort of patients treated with iodinated contrast was used as control. Baseline demographics and renal function tests were recorded. Primary outcome was incidence of contrast-induced nephropathy within 48–72 h post intervention. Receiver-Operating-Characteristic curve analysis was used to correlate the volume of iodinated contrast with the risk of contrast-induced nephropathy.

**Results:**

Carbon Dioxide was used as an alternative to iodinated contrast media in patients with impaired renal function (eGFR<60mls/min/1.73 m2) undergoing peripheral angioplasty procedures. Fifty, consecutive patients (baseline eGFR = 38.6 ± 13.2mls/min/1.73 m2) were included in a prospective clinical audit. These were matched (1:2) with a historical cohort of patients (baseline eGFR = 43.3 ± 12.2mls/min/1.73 m2) treated with Iodinated contrast media. The incidence of contrast-induced nephropathy was 14% (*n* = 7/50) in case of carbon dioxide vs. 29% (*n* = 29/100) in the matched cohort group (*p* = 0.045). Receiver-Operating-Characteristic analysis showed that use of >25mls of contrast was 94.4% (95% CI:81–99%) sensitive in predicting contrast-induced nephropathy.

**Conclusion:**

Carbon dioxide imaging during peripheral angioplasty procedures protects against contrast-induced nephropathy. Use of >25mls of iodinated contrast media in high-risk patients is a predictor of contrast-induced nephropathy.

## Background

Iodinated contrast media (CM) are amongst the most frequently prescribed medications worldwide today. They are used in a large number of both diagnostic and therapeutic procedures including but not limited to peripheral angioplasty procedures and percutaneous coronary interventions. Unfortunately, their use is not without complications. These may include allergic reactions, as well as contrast-induced nephropathy (CIN), especially in case of background chronic kidney disease (CKD). It is estimated that CIN accounts today for almost one third of all in-hospital acute kidney injury cases and present in 5–10% of coronary and peripheral interventions. Risk factors for CIN include diabetes mellitus, pre-existing renal impairment (CKD stage 3 or above, eGFR< 60 ml/min/1.73 m2 before intra-arterial administration or less than 45 ml/min/1.73 m2 before intravenous administration at baseline), age, heart failure, hypertension, anaemia and multiple myeloma (Shaw & Kessel, 2006). The estimated incidence of CIN is approximately 20% in case of pre-existing renal impairment. The in-hospital mortality after CIN is estimated around 7% in patients who will not require dialysis and as high as 35% in patients who will eventually require dialysis (Bansal, 2014).

To date, there have been several proposed strategies to reduce the incidence of CIN with most of them failing to prove their effectiveness in the clinical setting. CIN prophylaxis has been attempted with N- acetylcysteine and other agents with limited evidence. Our current practice for CIN prophylaxis is limited to intravenous hydration albeit with inconclusive effectiveness. Complete avoidance (by application of alternative diagnostic tests) or limitation of the overall volume of contrast may represent the most effective strategy in reducing the overall incidence of CIN (Briguori & Marenzi, 2006; Cigarroa et al., 1989; Isenbarger et al., 2003). Carbon dioxide (CO_2_) was introduced many years ago (1950s–1960s) as a non-iodine based alternative to contrast media for use during invasive angiographic studies either for diagnostic or therapeutic purposes. Its main advantage is that it is neither allergenic nor nephrotoxic making it safe to use in patients with either severe allergy to iodine CM or those suffering from renal impairment and where kidney protection is warranted (Fitridge et al., 1999; Hawkins et al., 2009; Nadolski & Stavropoulos, 2013). It may be given in unlimited amounts as it is rapidly cleared by the lungs, and is considered very safe for use under the level of the diaphragm.

The purpose of our study was to report our experience with the use of CO_2_ angiography in a number of peripheral arterial disease (PAD) patients and impaired renal function that underwent peripheral angioplasty or stenting and compare the incidence of CIN in this group with a historical cohort of CKD patients that underwent conventional angiographic procedures with the use of solely iodine CM.

## Methods

### Study design and population

We conducted a prospective clinical audit looking at the incidence of CIN in a number of patients with known renal impairment who underwent peripheral arterial interventions due to symptomatic peripheral arterial disease (PAD). The use of carbon dioxide (CO_2_) as an alternative contrast agent was formally initiated in patients with impaired renal function (eGFR<60mls/min/1.73 m2) undergoing peripheral endovascular interventions. All patients had both clinical assessment as well as a non-invasive imaging study (either Doppler ultrasound or non-contrast magnetic resonance angiography) before the procedure. During the 9-month study period, all patients suffering from critical limb ischemia (CLI) referred to the interventional radiology department for peripheral arterial intervention that were considered high risk for developing CIN, underwent either exclusively CO_2_ angiography or combined with supplementary use of small volumes of iodine CM which was recorded in detail and included in the analysis (CO_2_ group). Small volume of CM was administered in cases where images obtained with CO_2_ administration were inconclusive (degree and/or severity of post angioplasty dissection) to guide further treatment. Cases were matched (1:2) with a historical cohort of 100 consecutive patients treated solely with non-ionic low-osmolar iodinated contrast (Visipaque 320, Amersham; Control group). There were no exceptions made based on the arterial segment treated. This was a prospective service evaluation of carbon dioxide usage in peripheral interventions and a written informed consent prior to any intervention following detailed discussion of all the potential risks and benefits of CO_2_ use as an alternative to iodine-based contrast media. As per National Health Service Research and Ethics definitions (Institutional Review Board equivalent), this study is not classified as research and therefore formal ethics approval was waived.

Patients’ electronic medical records (electronic patient record-EPR, radiology information system-RIS) as well as relevant paper notes were retrospectively reviewed and analyzed for collection of baseline demographics, use of peri-procedure intravenous hydration and/or N-acetyl-cysteine (NAC). Baseline demographics included patients’ age at the time of the procedure, previous medical history including diabetes mellitus, hypertension, anemia and hypoalbuminemia. Cases that had received intravenous iodinated CM for other imaging investigations the week preceding the index angioplasty were excluded from inclusion in the present analysis. Patients under regular haemodialysis were also excluded from further analysis.

### Procedure

The use of CO_2_ for peripheral angiography has been described in detail previously (Funaki, 2008). All procedures were done in a dedicated Interventional Radiology suite (Artis Zee, Siemens, Erlangen, Germany) using the CO_2 –_Angioset (OptiMed, Ettlingen, Germany). For imaging optimization as recommended all patients had their legs elevated by 10–15 degrees usually by tilt of the angiographic table. Prior to CO_2_ administration all patients were lightly sedated with a combination of intravenous Fentanyl and Midazolam to minimize discomfort during CO2 injection. Care was taken to safely purge the CO_2_ set and avoid any air contamination prior to connection to the patient. Selective close-up angiograms were performed as necessary in order to optimize the image quality. The antegrade approach was preferred for femoropopliteal and tibial segments, while the ipsilateral retrograde (up-and-over) approach was employed for aorto-iliac disease. Injected CO_2_ volume was 60 mls for the iliac segment, 40 mls for the femoropopliteal segment and 20 mls for selective imaging of the below-the-knee arterial segments. In cases of inadequate opacification of an arterial segment, as per operator decision, iodine CM angiogram was performed and the total volume of iodine contrast used was recorded.

### Definitions, outcomes and statistical analysis

To date, there is no standard definition for reporting CIN. According to European Society of Urogenital Radiology (ESUR) guidelines, CIN may be defined as an increase in the creatinine (Cr) levels of more than 25% or 44 μmol (μmol)/L (0.5 mg/dl) compared to baseline within 3 days (72 h) following endovascular contrast administration (Guidelines E, 2015). Hence, this was adopted to for the purposes of this study to define the incidence of CIN (primary outcome). Secondary outcomes included differences (D) in serum creatinine values immediately and up to 30 days post procedure, total volume of iodinated CM used as well as calculation of a safe cut-off value of CM volume to be used in order to avoid CIN development based on receiver operating characteristics (ROC) curve analysis.

A univariate analysis of all potential covariates was performed in order to identify individual factors that may predict CIN development in the two study groups. These included patient age, diabetes mellitus, heart failure, hypertension, anaemia, CKD stages 4 or 5 (baseline eGFR < 30 mls/min/1.73 m2) versus CKD stage 3 (baseline eGFR > 30–59 mls/min/1.73 m2), hypoalbuminemia, total volume of carbon dioxide and finally total contrast volume below or above the cut-off value as calculated through the ROC curve analysis. Finally, any major complications associated with the use of CO_2_ were recorded. These included bowel ischemia, cardiac arrest, abdominal pain and nausea.

Statistical analysis was performed using the SPSS statistical software (SPSS, version 18.0 for Windows; SPSS Inc., Chicago, Il, USA.). Discrete and continuous variables are presented as counts and percentages, and as mean ± standard deviation respectively. Non-normal variables were expressed as medians and interquartile ranges (25th and 75th percentiles). The unpaired Student t test was used to identify statistical significant differences for variables that passed the normality test, while qualitative and continuous variables that did not pass the normality test were compared using the Mann-Whitney test. Receiver operating characteristics (ROC) curve analysis was used to identify the cut-off value of total CM volume in order to avoid CIN development.

## Results

### Study population and baseline data

During a 10-month period a total of 50 patients (mean age, 77.5 ± 10.4 years) underwent peripheral arterial interventions using either exclusively CO_2_ angiography or combined with supplementary use of small volumes of iodine CM (CO_2_ group). Cases were matched (1:2) with a historical cohort of 100 patients (mean age, 76.5 ± 10.5 years) treated solely with standard iodinated contrast (Visipaque 320, Iodixanol, GE Healthcare) (Control group) over a period of 19 months (June 2012 to December 2013). There were no statistically significant differences between the two groups with regards to diabetes mellitus, hypertension, heart failure, anemia, serum albumin levels, age (Table 1). The incidence of diabetes mellitus was approximately the same in both groups (66%, *n* = 33/50 and 64%, *n* = 64/100 for CO_2_ and control group respectively). Indication for treatment was CLI in the majority of cases in both groups and similar distributions of peripheral anatomical segments were revascularized involving mostly the superficial femoral and crural arteries without any significant comparative differences. All patients had baseline renal impairment (CKD 3–5), overall slightly more pronounced in the CO_2_ group but with similar baseline eGFR values. Similar incidence of CKD stages 4 or 5 (eGFR < 30mls/min/1.73 m2 at baseline) (24%, *n* = 12/50 CO_2_ Vs 21%, *n* = 21/100 control, *p* = 0.83) were observed in both groups. Patients’ baseline demographics, serum creatinine levels and eGFR values are shown in detail in Table 1.

**Table 1:** Baseline demographics and eGFR values data

Covariate	CO_2_ group	Control group	*P* value
Age (years)	77.5 ± 10.4	76.5 ± 10.5	0.88
Diabetes	33/50 (66%)	64/100 (64%)	0.4
Heart failure	13/50 (26%)	19/100 (19%)	0.07
Hypertension	36/50 (72%)	75/100 (75%)	0.34
Anemia	37/50 (74%)	84/100 (84%)	0.07
Hypoalbuminemia	28/50 (56%)	58/100 (58%)	0.41
Critical limb ischemia	39/50 (78%)	74/100 (74%)	0.74
Iliac arteries	9/50 (18%)	23/100 (23%)	0.62
Femoral arteries	30/50 (60%)	48/100 (48%)	0.23
Popliteal arteries	21/50 (42%)	39/100 (39%)	0.86
Tibial arteries	27/50 (54%)	58/100 (58%)	0.77
Creatinine baseline (μmol/Lt)	150.2 ± 48.8	136.5 ± 46.2	0.02
eGFR baseline (mls/min/1.73m^2^)	39.5 ± 11.6	43.0 ± 12.4	0.08
CKD 4 or 5 (eGFR< 30)	12/50 (24%)	21/100 (21%)	0.83
Intravenous hydration and/or NAC	42/50 (84%)	76/100 (76%)	0.36

### Outcomes

**Fig. 1:**
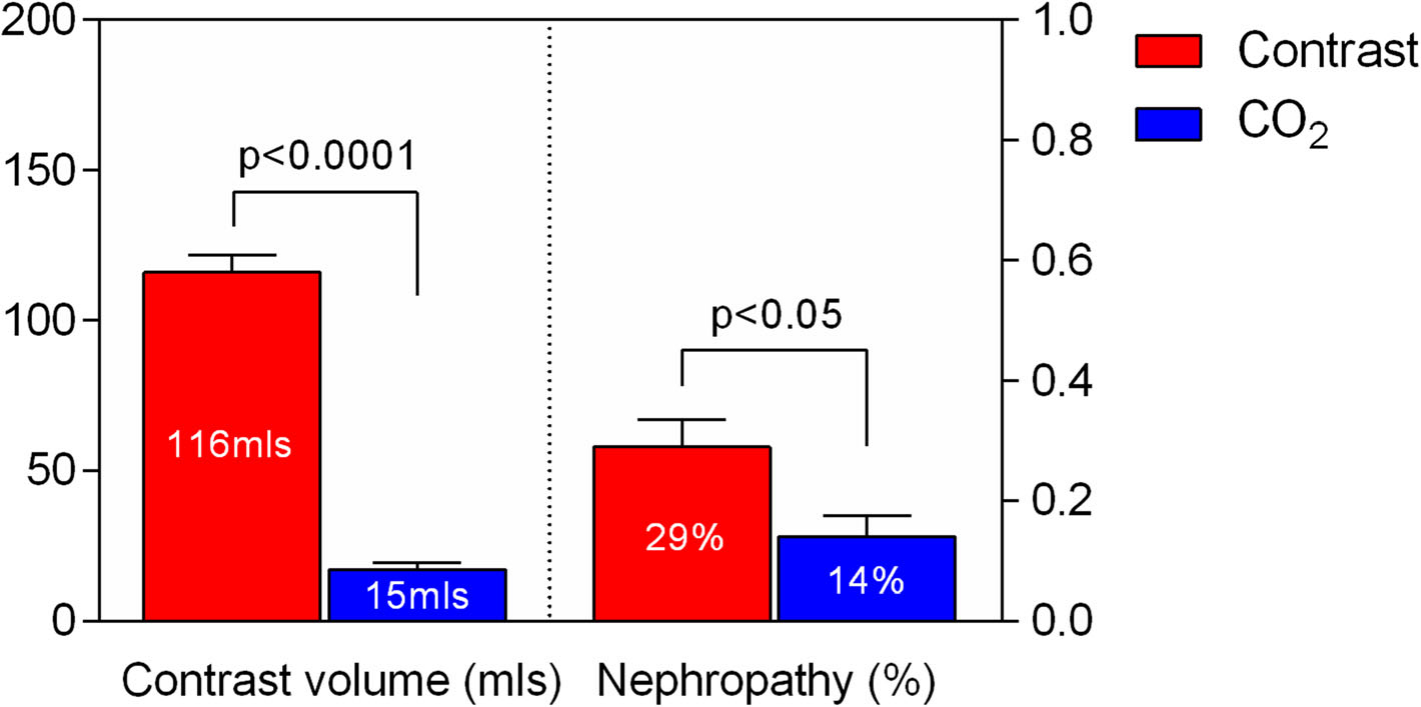
Total amount of iodinated contrast and incidence of CIN. CO2 (blue bars) and control (red bars) groups

There was a statistically significant difference in the overall incidence of CIN development post-procedure between the two groups. The rate of documented CIN was 14% (*n* = 7/50) in the CO_2_ group compared to 29% (*n* = 29/100) in the matched control group (*p* = 0.045). Similar numerical differences were noted in favour of the CO_2_ arm in the subset analysis between CKD stage 3 (eGFR between 30 and 60 mls/min/1.73m^2^) and CKD stages 4–5 (eGFR< 30 mls/min/1.73m^2^). Overall, the mean volume of iodine CM used was nearly 10 times greater in the control group compared to the CO_2_ group (115.6 ± 58.1mls Vs 15.1 ± 14.0mls, *p* < 0.001 respectively) (Fig. 1).

**Fig. 2:**
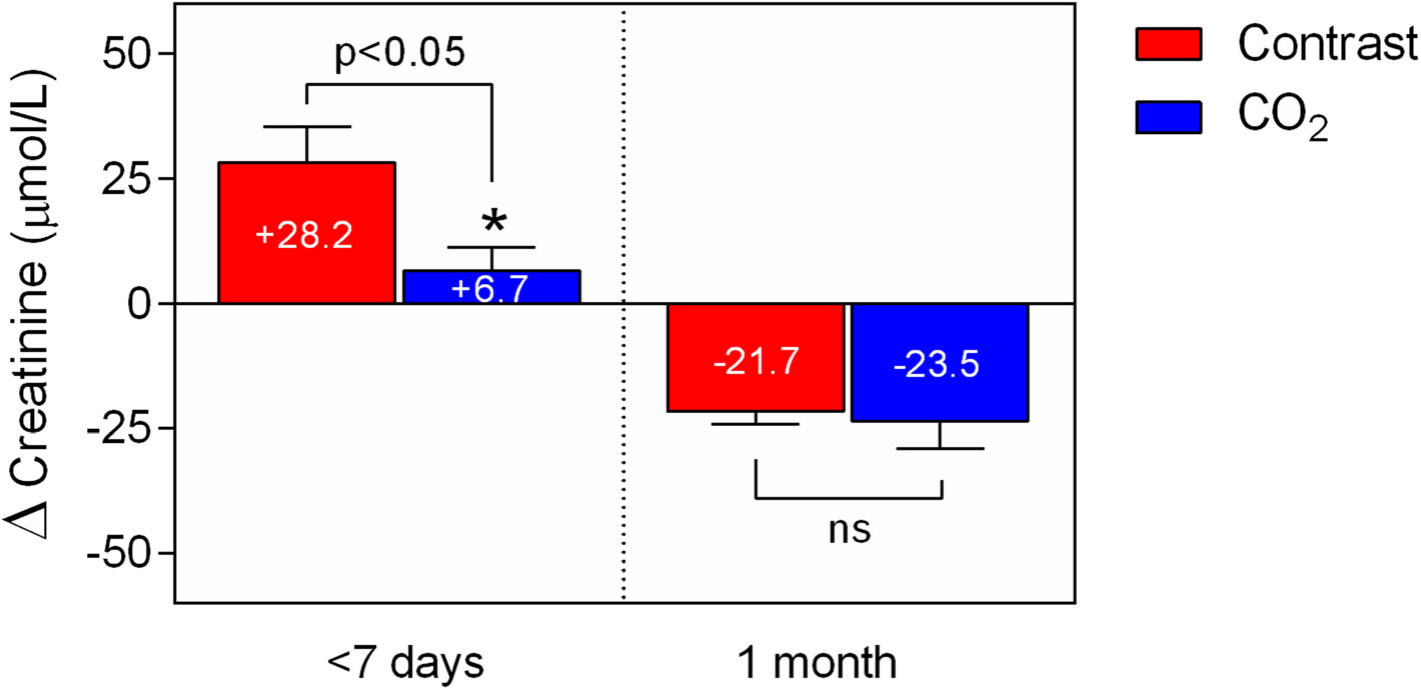
Serum creatinine differences (Δ) in the immediate post-procedure period and 30 days post-intervention. CO2 (blue bars) and control (red bars) groups

During the first post-procedure week there was a significantly greater rise in serum creatinine values in the control group (+ 28.2 ± 71.9 Vs + 6.7 ± 31.3 μmol/l in the CO_2_ group, *p* = 0.04). Interestingly, that difference was not evident at 30 days post-procedure when both groups demonstrated normalisation of the serum creatinine values compared to baseline (− 21.7 ± 26.0 in the control group Vs − 23.5 ± 38.5 *μ*mol/l in the CO_2_ group, p = ns) (Fig. 2).

**Fig. 3:**
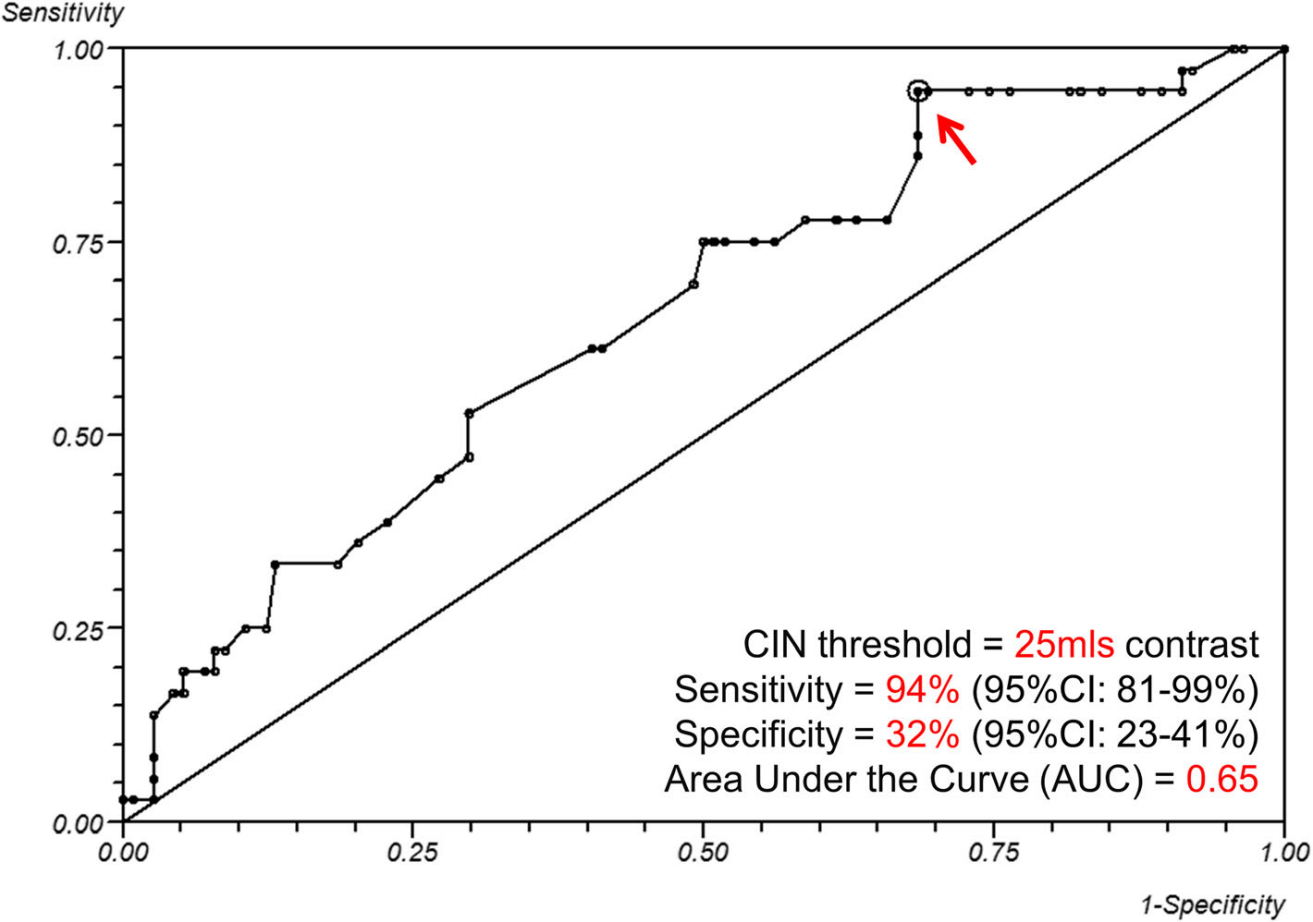
ROC curve of the volume of iodinated contrast. CIN cut-off value was 25mls of volume of iodine CM in predicting development CIN. [Sensitivity = 94.4% (95% CI: 81–99%), Specificity = 32% (95% CI: 23–41%), Area Under the Curve (AUC) = 0.65]

Receiver operating characteristic (ROC) curve analysis identified a cut-off value of 25 mls of iodine CM as a highly significant predictor for development of CIN during peripheral arterial interventions in patients with baseline renal impairment (Odds Ratio: 6.52, 95%CI: 1.48–28.8). The sensitivity was 94% (95%CI: 81–99%) with a specificity of 32% (95%CI: 23–41%), while the Area Under the Curve (AUC) was 0.65 (Fig. 3). The univariate analysis identified only the total volume of contrast as a significant predictor of CIN development; the risk of CIN was increased by 0.7 ± 0.3% per ml of iodinated CM administered. Results and univariate regression analysis of outcome predictors are shown in detail in Tables 2 and 3, respectively. There were no major complications associated with the use of CO_2_ in the present case series. Most of the CLI cases reported transient discomfort (seconds) at the level of the symptomatic foot that was well controlled with light conscious sedation.

**Table 2:** Clinical outcomes

Covariate	CO_2_ group	Control group	*P* value
Carbon dioxide	50/50 (100%)	0/100 (0.0%)	n/a
Contrast (mls)	15.1 ± 14.0	115.6 ± 58.1	< 0.001
CIN all cases	7/50 (14%)	29/100 (29%)	0.045
CIN in CKD 3	5/38 (13%)	22/79 (28%)	0.10
CIN in CKD 4–5	2/12 (17%)	7/21 (33%)	0.43
∆ creatinine value (*μ*mol/Lt) < 7 days	+ 6.7 ± 31.3	+ 28.2 ± 71.9	0.04
∆ creatinine value (μmol/Lt) at 30 days	-23.5 ± 38.5	−21.7 ± 26.0	0.94
Cut-off value of CM volume (as per ROC)	25 mls of iodinated contrast

**Table 3:** Univariate regression analysis of risk modifiers

Covariate	Odds Ratio	95% CI	*P* value
Age	0.99	0.95–1.02	0.52
Diabetes	1.51	0.66–3.45	0.33
Heart failure	1.42	0.59–3.46	0.44
Hypertension	1.11	0.45–2.72	0.82
Anaemia	1.90	0.61–5.92	0.27
CKD 4 or 5 (GFR < 30 mls/min/1.73m^2^ vs CKD 3 (30–59 mls/min/1.73m^2^)	1.35	0.56–3.27	0.51
Hypoalbuminemia	1.23	0.52–2.88	0.64
Contrast ≥25mls	6.52	1.48–28.8	0.01
Contrast volume	0.7 ± 0.3% per ml	0.2–1.3% per ml	0.009

## Discussion

Currently available strategies for CIN prevention including intravenous hydration (either alone or combined with n-acetylcysteine-NAC), administration of ascorbic acid, sodium bicarbonate, prophylactic hemofiltration have reported equivocal results. Practically, total avoidance or significant restriction of the overall volume of iodinated contrast seems to be the only effective strategy in reducing the CIN incidence (Briguori & Marenzi, 2006; Cigarroa et al., 1989; Isenbarger et al., 2003). Performing either diagnostic or therapeutic intra-arterial procedures using carbon dioxide is a well-known technique that was first described in the early 1900’s (Alexander, 2011). The main advantages of CO_2_ as a contrast media is the lack of iodine which makes it safe in patients with impaired renal function or patients allergic to iodine-based CM (Hawkins et al., 2009). Its main disadvantages are the transient discomfort during injection that requires the patients to be sedated during the procedure and the fact that it is contraindicated for use above the level of the diaphragm to prevent inadvertent coronary or cerebrovascular emboli.

In addition, there have been concerns of gas trapping in small branches of the celiac, superior or inferior mesenteric artery that can potentially lead to bowel ischemia. Logistical limitations include the need for specific gas set-up as well as the limited amount of total gas volume that can be used per time interval (no more than 100 cc of CO_2_ should be injected over a 2-min interval). CO_2_ angiography generally has somewhat inferior image quality due to the lower viscocity of the CO_2_ and motion artefact from transient pain during the injection can degrade the images further. All operators must be aware of the potential complications and take the appropriate precautions when using CO_2._ These include prevention of air contamination as carbon dioxide is colourless and therefore indistinguishable from air. Patients require monitoring to see if they develop abdominal pain, nausea, paraesthesia, leg pain or rarely accumulation and vapour lock of the pulmonary artery that may lead to hypotension and cardiac arrest (Moos et al., 2011).

Despite several reports highlighting the advantages and safety of CO_2_ angiography the method has not been widely accepted or used in everyday practice. Until recently, reports of CO_2_ angiography have been limited to small cohort studies reporting mostly its safety and diagnostic conspicuity. To our knowledge, this is the first comparative report that demonstrates compelling evidence about the reno-protective properties of carbon dioxide angiography in patients with chronic kidney disease. We have found that CO_2_ may effectively replace more than 90% of the amount of iodinated contrast that we use in everyday peripheral angioplasty procedures and thereby more than halve the incidence of contrast nephrotoxicity. Our findings are in line with some other sporadic cohort studies that sought to address the potential benefits of CO_2_ angiography.

Fujihara et al., observed a 5% CIN following CO_2_ angiography in a single-arm study of 98 patients (50% diabetics). The baseline eGFR was 35 mL/min and mean supplementary contrast volume used was 15 ml (Fujihara et al., 2015). Stegemann et al., recently showed a 5% CIN in the CO_2_ group (37 patients, 51% diabetics) Vs 29% in a control group of patients with normal renal function (154 patients, 51% diabetics) that received only iodine CM. The mean contrast volume in the CO_2_ group of this study was 34 ± 41 mls (Stegemann et al., 2016).

In our study the incidence of the CIN was slightly higher (14%) but significantly lower than the control group (29%). The mean supplementary iodine CM volume used in our study was 15.1 ± 14.0 mls. One reason for this difference can potentially be the higher number of diabetics in our series (68% in the CO_2_ group compared to 51% in the German study). In addition, our population involved predominantly patients suffering from more complex critical limb ischemia, whereas in the other two studies the study population was a balanced mix of intermittent claudication and CLI (Stegemann et al., 2016).

Another interesting finding in our study was the normalization of the creatinine levels in the majority of the cases in both groups within 30 days compared to baseline. Although in the immediate post-procedure period renal impairment deteriorated significantly more in the control iodinated contrast group, renal function markedly improved by day 30 compared to baseline in both groups and to the very same extent trending towards the low end of normal creatinine range. The authors attribute this to the high level of post procedure in-hospital care with vigilant monitoring of these critically ill patients, prompt supervision, use of CIN prophylaxis and guidance by responsible physicians involved in the care of this patient group in our institution.

### Limitations

The main limitation of this study is the inclusion of a historical control group for comparison purposes, as well as the relatively small number of patients included in the analysis. In addition, there were insufficient data about co-administration of other nephrotoxic medications that may have contributed to the renal dysfunction. Both groups have received appropriate CIN prophylaxis. A future study should assess CO2 angiography without CIN prophylaxis as this could improve efficiency for the service and convenience for the patient.

## Conclusion

In conclusion, CO_2_ is a valuable adjunct to iodinated contrast agents for evaluating and treating lower extremity arterial disease, minimizing the total volume of iodine contrast and thus preventing or reducing the overall incidence of CIN, especially in patients suffering from chronic kidney disease. Our study showed compelling evidence that the incidence of CIN was halved and renal function was largely maintained with use of CO_2_ compared to iodinated contrast medium. Finally, no more than 25mls of iodinated contrast should be allowed during angiography in high-risk patients. A future study should assess CO2 angiography without CIN prophylaxis as this could improve efficiency for the service and convenience for the patient.

## Data Availability

The datasets used and/or analysed during the current study are available from the corresponding author on reasonable request.

## Abbreviations

CIN: Contrast-induced nephropathy
CKD: Chronic kidney disease
CLI: Critical limb ischemia
CO2: Carbon dioxide
PAD: Peripheral arterial disease
ROC: Receiver operating characteristics
